# Some of the Therapeutic Effects of Caffeine When Hypodermically Administered

**Published:** 1886-09

**Authors:** 


### *SOME OF THE THERAPEUTIC EFFECTS OF CAFFEINE WHEN HYPODERMICALLY ADMINISTERED.*

The *Medical Record*, of July 17th, contains the experiments of Dr. John Cochrane, of Lowell, Mass., on the use of caffeine and its beneficial effects when administered hypodermically with morphine. He had sought a preventive for the depression and wretchedness which sometimes follows the exhibition of morphine, and found caffeine all that could be desired. In several of his patients morphine alone caused a depression amounting almost to collapse. In these same patients caffeine, given with morphine, sustained the pulvet nervous system in a remarkable manner without modifying the action of the morphine. The only unpleasant effect of caffeine so used is the thirst induced. He uses it in rheumatism and other diseases where there are signs of cardiac failure. He writes: "I have frequenty used this combination in cases of hysteria, of convulsions in infants and children who were teething or suffering from meningeal irritation, etc., with the best results. In acute alcoholic mania, when the heart begins to falter on account of the duration of the insanity and its concomitants, I cannot speak too highy either of its powers to stimulate the heart or of its calmative co-operation with morphia, while maintaining the nervous system, and so preventing dangerous symptoms.

Dr. Cochrane gives about the same dose of caffeine as of morphine, as shown by the following combination:

| | | |
|---|---|---|
| ℞ | Pulv. Caffeine, et., | |
| | Pulv. Morphia Sulph., - - - | aa gr. 1/6 |
| | Pulv. Atropiæ Sulph., - - - | gr. 1/100 |
| | Aq. Camp., - - - - - | M XX. |

